# Chemical analysis of Hg^0^-containing Hindu religious objects

**DOI:** 10.1371/journal.pone.0226855

**Published:** 2019-12-30

**Authors:** Adam M. Kiefer, Caryn S. Seney, Evelyn A. Boyd, Caroline Smith, Darran S. Shivdat, Elaina Matthews, Michael W. Hull, Christy C. Bridges, Amber Castleberry

**Affiliations:** 1 Department of Chemistry, Mercer University, Macon, Georgia, United States of America; 2 Analytical Instrumentation, Olympus Corporation of the Americas, Webster, Texas, United States of America; 3 Department of Biomedical Sciences, Mercer University School of Medicine, Macon, Georgia, United States of America; VIT University, INDIA

## Abstract

*Parad* items used in Hindu practices and Ayurvedic medicines contain elemental mercury (Hg^0^) and have traditionally been used in prayer and to treat a variety of diseases including diabetes, heart conditions, and sexual dysfunction. These items are often referred to as amalgams of silver, and take the form of *shivlings*, statues of gods, necklaces, and other jewelry. Fourteen *parad* items were purchased from online vendors in India and the United States and analyzed. All items produced copious amounts of Hg^0^ vapor, with Hg^0^ concentrations exceeding 1,000,000 ng/m^3^ as measured using a Mercury Instruments Mercury Tracker 3000 IP atomic absorption spectrometer. Measured concentrations were highly variable, so a simple qualitative experiment employing a UV-C light source and a thin-layer chromatography plate impregnated with a fluorescent dye that glows green when irradiated at 254 nm allowed for the indirect visualization of the Hg^0^ being evolved. In addition, all items were screened using a hand-held X-ray fluorescence analyzer to estimate the concentration of Hg, Sn, Pb, As, and Cd on the surface of the item. Select samples were then digested in *aqua regia* and analyzed for Hg content using a direct mercury analyzer. All samples were found to exceed 20% by mass Hg. The digestates were analyzed using inductively-coupled plasma–optical emission spectrometry and were determined to be between 10–55% by mass Pb and contain up to 0.3% by mass As. While Article 4 of the Minamata Convention on Mercury specifically requires parties to stop importing, exporting, and manufacturing Hg-added products, products used in traditional and religious practices are excluded.

## Introduction

Mercury and its related chemical compounds are potent human toxins, and are both persistent and transient in the environment [1–11]. In nature, mercury exists in its elemental form (Hg^0^), colloquially referred to as quicksilver; inorganic (Hg(I)/Hg(II) salts or complexes); or organic mercury, predominantly found as methylmercury (CH_3_Hg^+^). While the species of Hg dictates its toxicity, all mercury species are toxic and are in a complex equilibrium known as the Global Mercury Cycle [8,9]. The organ systems affected by mercury vary based upon the chemical composition of the toxin and how it is introduced into the human body. For example, Hg^0^ is of limited bioavailability as a liquid, but when inhaled Hg^0^ vapor is rapidly converted to Hg^2+^ in the blood stream. Chronic exposure to Hg^0^ vapor leads to erethism, historically referred to as “Mad Hatter’s Disease” for the haberdashers that presented with tremors, confusion, behavioral changes, delirium, memory loss and kidney damage as a result of exposure to Hg^0^ [12]. At higher concentrations, mercurial pneumonitis and death can result [4]. Unlike Hg^0^, inorganic mercuric (Hg^2+^) and mercurous (Hg^+^) salts and complexes are nonvolatile and are toxic via ingestion. These water-soluble complexes are readily absorbed by the gastrointestinal tract and are highly nephrotoxic and neurotoxic. Inclusion of calomel (Hg_2_Cl_2_) in skin lightening creams has led to incidents of poisonings via dermal uptake, inhalation, and possible ingestion [13–16]. Recent evidence also indicates that when calomel is used on human skin, reduction to Hg^0^ can occur, increasing the rate of contamination throughout households [13]. The highly toxic organometallic species CH_3_Hg^+^ is produced by anaerobic bacteria and rapidly bioaccumulated and biomagnified in the environment. Most human exposure to CH_3_Hg^+^ is typically via ingestion of food, particularly carnivorous fish [17–19]. Exposure to organomercury species is known to cause “Minamata Disease”, which includes neurological impairment and kidney damage [12]. Due to the toxicity, persistence, and mobility in the environment of mercury and its related compounds, the Minamata Convention on Mercury was written and signed by over 120 nations [20]. This International Treaty attempts to curtail mercury use, thus limiting anthropogenic mercury pollution and human exposure [21–26]. The convention, which entered force in August of 2017, ultimately bans the mining of primary mercury ore and severely restricts the use of mercury in industry and in mercury-added products.

In spite of their toxicity, mercury-added products used for traditional and religious purposes have been historically used in traditional medicines and religious practices [27–41]. Riley and coworkers note that in Latino and Caribbean communities Hg^0^, referred to as *azogue*, is used to ward off evil spirits, bring luck, lead to love or money, and treat intestinal disorders [36]. It is also noted that it is often difficult to differentiate between religious and cultural uses of mercury in these communities [36]. Hg^0^ has been reportedly used in amulets, sprinkled on floors and in automobiles, and placed in a variety of household items to spiritually cleanse a dwelling [35]. These actions have led to highly elevated Hg^0^ concentrations in residential areas, and improper use and storage has resulted in exposure of children to high concentrations of vapor necessitating chelation therapy [39,42]. In addition, traditional Chinese medicine has employed the use of Hg for millennia, and the inclusion of cinnabar (HgS) in medicines has recently been reported [34,43]. Mercury (II) oxide (HgO), mercury (II) chloride (HgCl_2_), and HgS are also used in Ayurvedic medicines, a holistic medical practice originating in India [28,32,40,41]. In one instance Hg^0^ and sulfur are claimed to be detoxified by the addition of a variety of chemicals, garlic and milk. The Hg^0^ is mixed with an excess of elemental sulfur to form HgS, and sublimed [31]. While significantly less bioavailable than other forms of Hg, HgS has been demonstrated to effect a variety of organ systems in mouse models [44,45].

**Fig 1:**
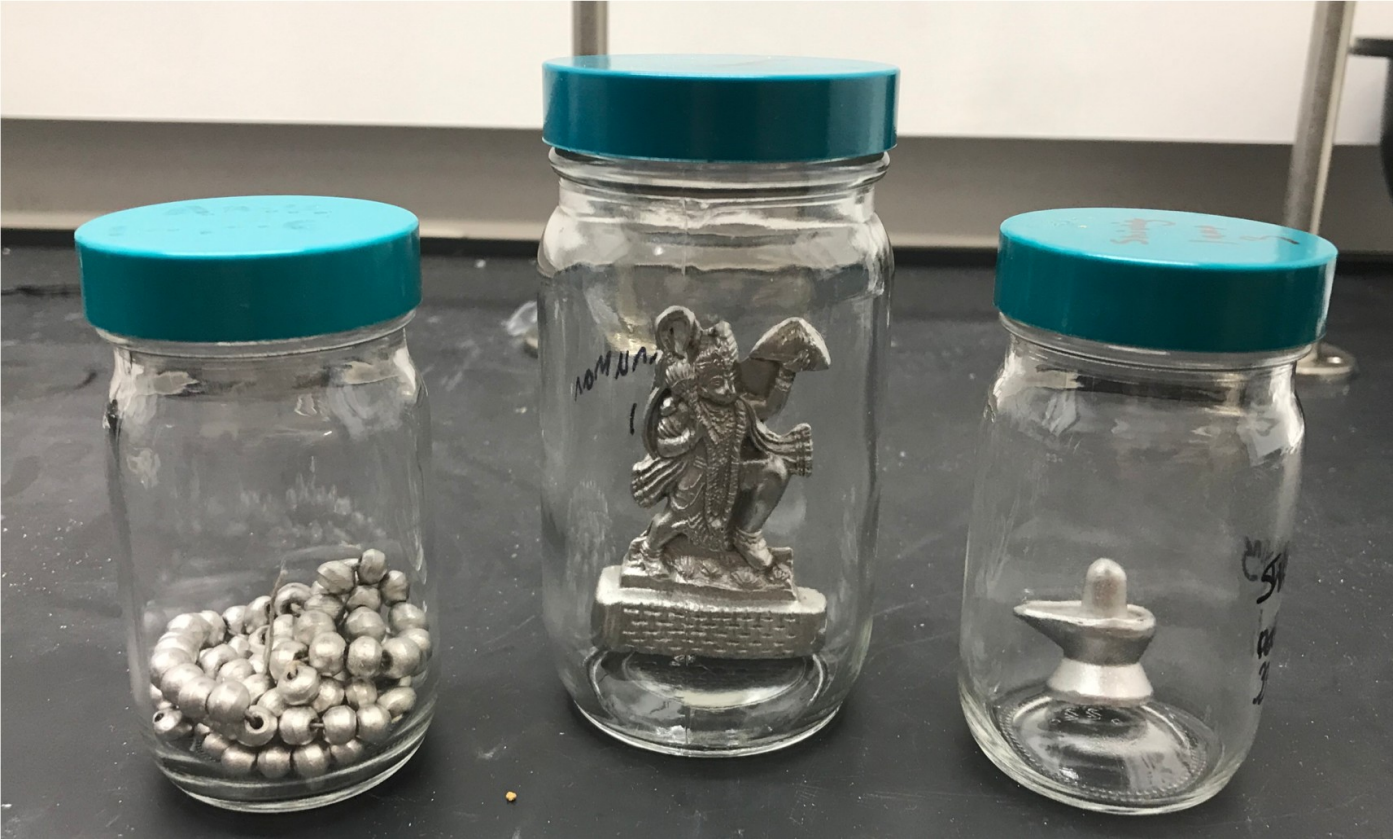
Amalgams can be molded into a variety of shapes including *malas* (necklaces), deities, and *shiva lingams* (L→R).

Hg use in Ayurveda has been documented over thousands of years, and the use of Hg^0^ overlaps with the Hindu religion and other cultural practices. Hg^0^, or *parad*, is important in the Hindu religion and is representative of the sperm of Lord Shiva [34]. When mixed with other metals, Hg^0^ forms a solid alloy referred to as an amalgam; during the amalgamation process the amalgam can be shaped and molded into items of religious meaning such as *lingas*, which are representative of the phallus of the Lord Shiva. Additionally, the amalgam can be molded into *sri yantras*, statues of other deities, necklaces, rings, and cups for drinking (Fig 1) [34,46]. Traditionally, *parad shivlings* were constructed from silver and mercury with the inclusion of herbs and vegetal material in order to render the Hg^0^ nontoxic. These amalgamated objects have use in religious worship and in addressing illnesses. Wearing *parad* objects is claimed to cure or treat diabetes, high blood pressure, heart disease, pain, digestive issues, marriage problems, and nightmares. They are also thought to protect from evil spirits. The *parad* item affords these protections either via wearing the item, placing the item in direct contact with the afflicted site, or via pouring unboiled cow’s milk over the item and drinking the milk [46].

Unlike Hg-containing Ayurvedic medicines, the preparation and stability of *parad* items is not well described in the peer-reviewed literature, nor have any studies directed towards their safety and stability been conducted. Specifically, the emission of Hg^0^, which is invisible to the naked eye, represents a real threat to human health. Numerous *parad* items were purchased online from distributors in the United States and India and analyzed using atomic absorption spectroscopy, X-Ray Fluorescence, Direct Mercury Analysis (DMA-80), and Inductively Coupled Plasma–Optical Emission Spectroscopy (ICP-OES). A simple system involving a Hg^0^ lamp and commercially available thin-layer chromatography (TLC) plates allowed for the visualization of Hg^0^ vapor in real time. In addition to conventional chemical analysis of the amalgams, a simple means of demonstrating that Hg^0^ vapor is emitted from these objects may be beneficial in teaching practitioners to use *parad* items safely, or to abandon their use altogether.

## Health and safety

All *parad* items analyzed in this paper were handled using nitrile gloves in either a fume hood or under a fume extractor. Each object contained quantifiable amounts of toxic metals including Hg, Pb, and As. In particular, every item emitted Hg^0^ vapor. The amount of vapor liberated increased during the handling of the object. For these reasons, objects were handled infrequently, and stored in glass jars located in a fume hood. Since *aqua regia* is a strong, oxidizing solution and was produced and handled in a fume hood, as were all chemical digestions. All solutions were disposed of following appropriate waste protocols.

## Materials and methods

### Chemicals and reagents used

A trace metal grade mercury standard stock solution (1000 μg/mL, 5% nitric acid) was purchased from Alfa Aesar (Cat No. 88079). Mercury standards were made through the subsequent dilution of the stock solution in order to generate calibrations curves for the DMA-80 instrument. An additional standard stock solution was purchased from Perkin-Elmer (26 multi-element Std Pure: PE No. N9301721). Lead (Pb), arsenic (As), cadmium (Cd), and chromium (Cr) standards were prepared through subsequent dilution of the stock solution to generate calibration curves for the ICP-OES instrument. A mercury in water standard reference material (NIST SRM 1641d) was purchased from Sigma Aldrich. Trace metal basis (99.999%) nitric acid (HNO_3_) was purchased from Sigma-Aldrich.18.2 MΩ*cm ultrapure water was obtained from a Siemen’s ELGA LabWater purification. *Parad shivlings*, idols and necklaces were purchased from online sellers in the United States and India through amazon.com and ebay.com; they were found using the keywords “parad shivling” or “parad statue”. Descriptions of these items, including photos, are available in S1 File. Samples not digested during the course of investigation are available upon request from the authors. All items were unpackaged in a fume hood and sealed in glass jars. Silica G Thin Layer Chromatography plates w/ UV 254 + 366 plates were purchased from Sorbent Technologies and irradiated with a UVP Model UVG-11 lamp at 254 nm.

### Sample preparation

The *parad* items analyzed in this paper were difficult to manipulate in the laboratory. Cutting items was neither practical, nor safe as the friction from the blade would result in contamination of the surrounding area with Hg^0^. In addition, the *parad* items are exceptionally hard and difficult to break into smaller pieces. Therefore, a selection of *parad* items were wholly digested in *aqua regia* (3 parts of HCl: 1 part HNO_3_). The digestion method was developed using individual beads from the *parad* necklace. Upon inspection, the individual beads were uniform in color, luster, size, shape, and weight (~1g/bead). The one g beads were individually digested in 50 mL of the *aqua regia* solution within a few hours with gentle heating to 50°C. The 9.9564 g *shivling* was heated to 50°C for one hour in 500 mL of *aqua regia* and cooled, dissolving overnight. The amount of *aqua regia* required to fully digest the 38.0556 g statue was deemed to be unsafe. The item was frozen in liquid nitrogen, placed in a Ziploc bag, and three pieces weighing 1.0071 g, 2.1010 g and 3.7391 g were removed using locking pliers. Each of these three pieces required ~300 mL:1 g *aqua regia* : *parad* with heating to 200°C over three days. It is assumed that the rate of dissolution is dependent upon the relative concentrations of metals present in the sample. After digestion, the mass of the digestate was recorded. For analysis these samples were then diluted with 18.2 MΩ*cm ultrapure H_2_O in order to ensure that measured concentrations were within the range of the calibration curve.

### Instrumentation

A Milestone Direct Mercury Analyzer was used for the determination of mercury in aqueous samples. This single beam spectrophotometer relies on thermal decomposition of the sample and a catalytic reduction of all mercury to Hg^0^, amalgamation, and atomic absorption spectrometry. 100 *μ*L samples were added to quartz boats and analyzed via the DMA-80 Dual Cell. The DMA-80 was automated and controlled by Windows Easydoc 3.3 software. All samples were analyzed in triplicate. Lead, arsenic, cadmium and chromium levels in aqueous samples were determined using a Perkin Elmer (PE) Optima 8300 Concentric ICP-OES equipped with a SC-4 DX Autosampler. Samples were introduced using a sea spray nebulizer with a cyclonic spray chamber. The Optima 8300, the autosampler and peristaltic pump were fully automated and controlled by the Windows ICP Syngistix Controller software. All samples were analyzed in triplicate. Operating details relating to data collection and analysis are available in the supporting information (S2 File). Air concentrations of Hg^0^ were measured in real time using a Lumex RA-915M (Lumex) and a Mercury Instruments Mercury Tracker IP (MTIP). These portable atomic absorbance spectrometers are tuned to 253.7 nm, and utilize a pump to draw ambient air through the instrument. The instruments determine Hg^0^ in real time. Both instruments were calibrated by the manufacturer. The Lumex is calibrated to monitor concentrations ranging from 0–38,959 ng/m^3^, and the MTIP is calibrated to monitor concentrations from 0–2,000,000 ng/m^3^. Chemical analysis of the surface was conducted using an Olympus Vanta C Handheld XRF analyzer with a field stand kit. Samples were analyzed using the manufacturer’s GeoChem(2) and AlloyPlus methods in triplicate. The instrument calculates 3-σ as error; as a result, samples detected below 3-σ return an N.D. (not detected). Both calibration methods utilize a fundamental parameters calculation method [47–51]. Portable X-Ray Fluorescence analyzer (Olympus Vanta C Series) does not require the amalgam to be placed under vacuum, which would risk contaminating or damaging the instrument with volatilized Hg^0^. The silica G Thin Layer Chromatography plates w/ UV 254 + 366 plates were used to visually show the volatility of the *parad* samples.

## Results and discussion

### Qualitative assessment and analysis of Hg^0^ vapor emitted by *parad* objects

The *parad* objects were received via the United States Postal Service. Received items were dull grey to silver in color. Items shipped from India were received in a small synthetic pouch, wrapped multiple times in plastic and then in cloth. The item was then placed in a cardboard box, and the box was wrapped in hand-sewn cloth. Items shipped from within the United States were placed either in a plastic case wrapped in bubble wrap; wrapped directly in bubble wrap; or wrapped in cotton, then bubble wrap. They were then placed in a 2-day air shipping envelope. Measurements of Hg^0^ vapor were collected during the unwrapping process of samples received from the United States. The packages were placed under a fume extractor, where ambient Hg^0^ was previously measured as being below 0.010 μg/m^3^ using a Lumex RA-915M. Two of the shipped packages registered measurable Hg^0^ concentrations within 30 cm of the package. Concentrations of Hg^0^ within 5 cm of the outside of the packaging ranged between 0.100–0.500 μg/m^3^ when they were not disturbed. During the opening process, a small tear was made in the packaging and concentrations rapidly exceeded 6 μg/m^3^ within 15 cm of the packages but were highly fluxional. When gentle pressure was placed upon the outside of one shipping envelope during the opening, concentrations increased dramatically, exceeding 18 μg/m^3^, at which point the Lumex was removed to prevent saturation of the detector and/or contamination of the instrument. The packages were sealed and transported to a fume hood. Concentrations during the remainder of the unwrapping were monitored using the MTIP. Continued monitoring during the unwrapping process was complicated due to the high air flow through the fume hood; however, when the final bubble wrap was removed from one item the MTIP recorded concentrations exceeding 350 μg/m^3^ in proximity to the object. During the final stage of unwrapping, all objects exceeded 250 μg/m^3^ as measured by the MTIP.

**Fig 2:**
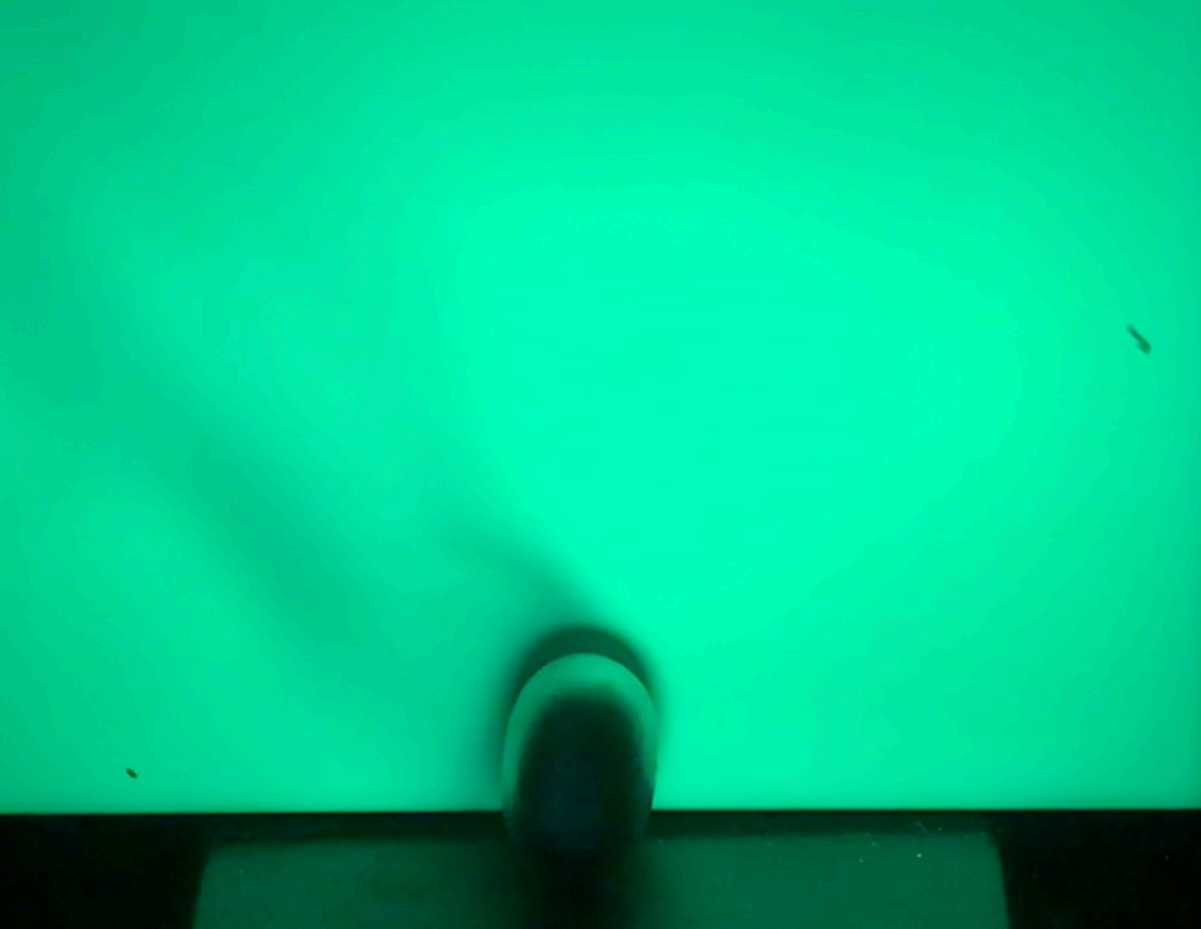
A *parad shivling* is placed in front of a TLC plate and irradiated with light from a lamp emitting at 254 nm. The light is absorbed by the Hg^0^ vapor emitted from the *shivling*, casting a dark shadow on the fluorescing TLC plate.

Mercury concentrations measured around *parad* objects are highly fluxional. The MTIP measures a sample every second, and over short time spans concentrations will fluctuate from a few μg/m^3^ to concentrations exceeding 1,000 μg/m^3^. To understand the origins of this fluctuation, a simple qualitative experiment was devised to indirectly visualize Hg^0^ vapor emitted from the amalgams. Each amalgam was placed in front of a vertical TLC plate containing a dye that fluoresces green when irradiated at 254 nm, the same wavelength of light that Hg^0^ strongly absorbs. When irradiated with a UV-C lamp emitting light at 254 nm, the vapor absorbs the light, casting a shadow on the bright green TLC plate allowing for the visualization of emitted vapor (Fig 2). The stream of Hg^0^ emitted was observed to be easily affected by disturbances in the air around the amalgam and rapidly dissipates as it becomes diluted in the atmosphere; this leads to dramatic differences in concentrations measured by the MTIP and Lumex spectrometers. In addition, the Hg^0^ vapor appears to be too dense in bulk to be transported through the sampling probe to the MTIP spectrometer, indicating that measured Hg^0^ concentrations may be significantly lower than in reality. An example of a standard visualization experiment coupled with air analysis using the MTIP is available on YouTube [52].

All *parad* items emitted Hg^0^ vapor as measured by the Lumex and MTIP and indirectly visualized by the aforementioned method. When placed in front of the screen all items emitted copious amounts of Hg^0^, and although over time the visible plume diminished, Hg^0^ was still spectroscopically measured. The decrease in visual emissions was attributed to the oxidation of metals on the surface of the amalgam, forming an oxide layer temporarily limiting the volatilization of Hg^0^ [53–58]. Importantly, any physical contact with the surface of the amalgam increased concentrations of Hg^0^ in the air. Manipulation of all amalgams with a gloved hand led to the transfer of the amalgam to the glove as a silver/grey solid. While this caused the *parad* items to emit additional Hg^0^, it resulted in significant amounts of Hg^0^ being emitted from the glove that exceeded the 2,000 μg/m^3^ detection limit of the MTIP.

### Chemical analysis of the surface of *parad* items via X-Ray fluorescence

Hg^0^ vapor is likely emitted exclusively from the surface of the *parad* items. To date, there have been no thorough studies of other metals that are present in these amalgams. A rapid screening of the metals present on the surface would be useful in ascertaining which metals should be determined by rigorous quantitative techniques. This rapid screening is even more valuable considering that the presence of certain metals has been demonstrated to affect the volatilization of Hg^0^ from the surface of amalgams [58,59]. The risk of contamination was compounded by the fact that even gently handling the items seemingly transferred Hg^0^ to the surface of nitrile gloves. Subsequent analysis of the gloves via XRF demonstrated that Pb and As were also transferred to the glove, indicating that the amalgam itself is transferred to the glove. The analysis of the surface of the amalgams for Cr, As, Ag, Cd, Sn, Hg, and Pb is found in Table 1.

**Table 1:** Analysis of *Parad* Items by XRF (% ± SD).

Item	Mass (g)	Method	%Hg	%Pb	%As	%Cd	%Cr	%Sn	%Ag
**Lingam 1**	105.9106	AlloyPlus	--	19.17 ± 0.37	--	N.D.	N.D.	42.64 ± 1.41	N.D.
		Geochem(2)	19.29 ± 0.35	12.96 ± 0.39	2.12 ± 0.064	N.D.	0.12 ± 0.012	34.77 ± 0.50	N.D.
**Lingam 2**	118.6880	AlloyPlus	--	19.42 ± 0.45	--	N.D.	N.D.	41.92 ± 0.75	N.D.
		Geochem(2)	19.44 ± 0.13	12.91 ± 0.14	2.07 ± 0.022	N.D.	0.12 ± 0.0059	35.46 ± 0.35	N.D.
**Shivling 1**	9.9564	AlloyPlus	--	6.87 ± 0.43	--	N.D.	N.D.	50.23 ± 3.11	N.D.
		Geochem(2)	21.96 ± 1.43	5.32 ± 0.32	0.88 ± 0.072	N.D.	0.11 ± 0.0078	43.27 ± 1.87	N.D.
**Shivling 2**	38.8661	AlloyPlus	--	33.42 ± 2.20	--	N.D.	N.D.	28.09 ± 1.45	N.D.
		Geochem(2)	24.76 ± 0.42	20.25 ± 0.74	3.14 ± 0.086	N.D.	0.13 ± 0.0090	23.46 ± 0.75	N.D.
**Lingam 3**	105.8539	AlloyPlus	--	15.76 ± 2.26	--	N.D.	N.D.	46.63 ± 3.62	N.D.
		Geochem(2)	18.56 ± 0.57	11.88 ± 1.58	1.89 ± 0.29	N.D.	0.12 ± 0.014	36.21 ± 1.39	N.D.
**Shivling 3**	38.6787	AlloyPlus	--	31.24 ± 0.31	--	N.D.	N.D.	28.52 ± 2.00	N.D.
		Geochem(2)	23.89 ± 0.65	18.90 ± 0.86	2.89 ± 0.13	N.D.	0.13 ± 0.018	22.89 ± 0.79	N.D.
**Shivling 4**	21.8004	AlloyPlus	--	28.69 ± 3.71	--	N.D.	N.D.	24.35 ± 0.69	N.D.
		Geochem(2)	27.20 ± 1.59	18.21 ± 1.83	2.84 ± 0.26	N.D.	0.12 ± 0.0088	21.64 ± 1.33	N.D.
**Shivling 5**	21.6029	AlloyPlus	--	31.40 ± 4.60	--	N.D.	N.D.	25.57 ± 0.90	N.D.
		Geochem(2)	27.82 ± 1.50	18.48 ± 1.40	2.88 ± 0.26	N.D.	0.13 ± 0.013	21.51 ± 1.03	N.D.
**Pyramid**	62.7012	AlloyPlus	--	27.22 ± 2.04	--	N.D.	N.D.	27.66 ± 0.55	N.D.
		Geochem(2)	29.31 ± 1.29	16.45 ± 0.38	2.60 ± 0.087	N.D.	0.12 ± 0.0051	24.34 ± 0.85	N.D.
**Hanuman**	137.64^*a*^	AlloyPlus	--	49.02 ± 2.09	--	N.D.	N.D.	23.21 ± 1.97	N.D.
		Geochem(2)	20.73 ± 0.34	27.43 ± 0.46	3.47 ± 0.11	N.D.	0.14 ± 0.017	19.65 ± 0.61	N.D.
**Statue 1**	38.0566	AlloyPlus	--	49.10 ± 0.20	--	N.D.	N.D.	25.10 ± 0.86	N.D.
		Geochem(2)	19.91 ± 0.08	27.78 ± 0.47	3.34 ± 0.016	N.D.	0.15 ±0.024	20.72 ± 0.15	N.D.
**Statue 2**	19.5109	AlloyPlus	--	27.91 ± 3.68	--	N.D.	N.D.	47.89 ± 3.59	N.D.
		Geochem(2)	14.66 ± 0.31	17.59 ± 2.56	1.70 ± 0.35	0.14 ± 0.014	0.15 ± 0.024	35.72 ± 2.65	N.D.
**Statue 3**	33.7205	AlloyPlus	--	49.54 ± 1.77	--	N.D.	N.D.	24.16 ± 1.67	N.D.
		Geochem(2)	19.56 ± 0.92	27.11 ± 2.06	3.33 ± 0.29	N.D.	0.15 ± 0.024	20.88 ± 1.23	N.D.
Necklace (Beads)	~1 (per bead)	AlloyPlus	--	4.23 ± 0.30	--	N.D.	N.D.	65.11 ± 0.85	N.D.
		Geochem(2)	15.22 ± 0.59	3.36 ± 0.45	0.59 ± 0.076	N.D.	0.12 ± 0.0093	51.25 ± 2.25	N.D.

^*a*^ measured on a top loading balance; N.D.: Not Determined

Handheld XRF analyzers have been used to great effect to analyze materials for heavy metal contamination, and have been employed to rapidly screen materials to ensure compliance with the Restriction on Hazardous Substances Directive (RoHS) [60,61]. While amalgams are by definition alloys, they have unique physical and chemical properties that are uncommon in most conventional materials. The XRF employed to screen amalgams is not calibrated by the user, but rather utilizes methods that are designed for specific applications and installed by the manufacturer. Each *parad* sample was analyzed in triplicate using two different methods: AlloyPlus and GeoChem(2). Both calibrations employ a fundamental parameters methodology for calculating the chemical weight percent of the sample. Fundamental parameters calibrations attempt to provide “total chemistry”, and as such normalize to 100% [47–51]. As the name implies, the AlloyPlus method is designed to identify commercially available alloys, and as such, does not measure Hg. The manufacturer’s AlloyPlus calibration assumes the absence (in any appreciable amount) of any elements lighter than magnesium in the sample (e.g., carbon or oxygen). In other words, the AlloyPlus calibration assumes that all metals are present in their metallic (non-valent) state. In contrast, the GeoChem(2) method assumes the presence of elements lighter than magnesium, most commonly oxygen. Therefore, it is often used for screening soil, mineral, geological, and other low-density samples [62]. Previous work has indicated that the surfaces of amalgams are prone to evaporation of Hg^0^ and oxidation of the non-volatile metals, and as such this method may yield a reasonable approximation of metal concentrations on each *parad*. The specimens were measured with both methods, and comparative data is shown in Table 1. It is important to note that while XRF can be a quantitative analytical technique, the results presented herein are not intended to be quantitative.

As expected, screening with the XRF indicated that a significant portion of each *parad* item was Hg. However, the measurable amount of As found was unexpected. Although many online sources state that *parad* items contain Ag, none was detected using either method—the AlloyPlus or Geochem(2). The major component of most items was Sn, as was reported in an initial screening the environmental non-governmental organization Toxics Link [46]. It was not surprising that Pb was found in each item; however, the dramatic difference in concentrations of Pb as measured by the AlloyPlus and Geochem(2) methods was noted. Again, the XRF was intended to be used as a preliminary screening technique to identify the presence of certain metals, and no effort was made to refine the methods to quantify metal content using the XRF. Based upon this initial screening, samples procured from different manufacturers were selected for further analysis to determine the concentrations of Hg by DMA-80 and Pb, As, Cd, and Cr by ICP-OES.

### Determination of Hg, Pb, As, Cd and Cr by DMA-80 and ICP-OES

The formulation and preparation of *parad* items is not well described in the literature. As a result, there is no way to determine if a homogeneous amalgam is first formed and compressed in a mold to form the *parad* object, or if an object made from tin, lead, or an inexpensive alloy has mercury incorporated onto its surface to appear to be an amalgam. Because XRF analysis is only a surface technique, it cannot exclude the latter procedure. Similarly, an object in which only the surface was amalgamated could still emit copious amounts of Hg^0^ vapor. While cutting into *shivlings* to analyze the center of each item via XRF was initially considered, even small amounts of physical contact with the surface released dangerous concentrations of Hg^0^ vapor. It was assumed that the friction associated with sawing through the amalgam would release Hg^0^, contaminating the fume hood. Based upon the aforementioned video evidence, there was concern that the fume hood would not be able to remove the Hg^0^ as it was being produced. Therefore, it was decided that *parad* items would be digested, whole or in part, to determine if the items were an amalgam or if only the surface was amalgamated.

The digestates were diluted and analyzed via direct mercury analysis and ICP-OES (Table 2). Cd and Cr were found to be below the limit of detection (LOD) for the ICP-OES in agreement with the XRF data. Again, it is important to note that the XRF used in this study is not calibrated by the operator and relies on pre-established methods. The GeoChem(2) method estimates the percentage of oxidation in the sample, while the AlloyPlus method omits Hg and As because they are rarely found in commercial alloys. As a result, Hg and As were only estimated by XRF using the Geochem(2) method. Every item was routinely estimated by the XRF to be lower in percent Hg than was determined by the DMA-80, with percent agreements of 72–99%. The results clearly indicate that the entirety of the *parad* objects contained Hg°. This supports the assertion that an amalgam was prepared and compressed into a mold to generate the object as opposed to only amalgamating the surface of the object. Estimates of the % As by XRF were 410%-1500% higher than the concentration as determined by ICP-OES, but importantly the XRF still indicated the presence of As in the sample.

**Table 2:** Determination of Hg, Cd, Cr, Pb, and As in select *parad* objects (% ± SD).

Item	%Hg	%Pb	%As	%Cd	%Cr
**Bead (1)**	20.87 ± 0.19	9.799 ± 0.002	0.1429 ± 0.0033	< LOD	< LOD
**Bead (2)**	21.03 ± 0.17	10.00 ± 0.00	0.1250 ± 0.0032	< LOD	< LOD
**Bead (3)**	20.31 ± 0.95	10.62 ± 0.00	0.1350 ± 0.0016	< LOD	< LOD
**Shivling 1**	23.63 ± 0.11	9.532 ± 0.002	0.1012 ± 0.0041	< LOD	< LOD
**Statue 1 (a)**	20.72 ± 0.68	52.84 ± 0.00	0.3158 ± 0.0020	< LOD	< LOD
**Statue 1 (b)**	20.19 ± 0.31	54.91 ± 0.00	0.2172 ± 0.0040	< LOD	< LOD
**Statue 1 (c)**	20.22 ± 1.22	44.17 ± 0.00	0.2208 ± 0.0042	< LOD	< LOD

The over-reporting of arsenic is likely due to high levels of lead in the sample. The Pb L_α_ (10.55 KeV) peaks overlaps with the arsenic K_α_ (10.54 KeV) peak. At low-to-moderate concentrations of lead, fundamental parameters methodologies generally resolve this interference fairly successfully. But at higher concentrations accurate deconvolution become more challenging [63,64]. Interestingly, Pb levels, as determined by ICP-OES, were higher than reported by the XRF. Employing the AlloyPlus method of the XRF in all cases resulted in better agreement to the data collected by ICP-OES, resulting in a range of agreement from 44–110%. XRF may prove to be a valuable tool for rapidly identifying the presence of these highly toxic elements in *parad* objects, but accurately quantifying the amounts in individual items must be done using more rigorous digestion methods and more analytically sensitive instrumentation.

### Potential ramifications for human health

In spite of the claim that the purification involved in the preparation of *parad* items used in Hinduism and Ayurvedic medicine renders these items safe for handling and storage in the home, chemical analysis of these items indicate that they may represent a real threat to human health. Concentrations of Hg^0^ measured in the air immediately surrounding each item is orders of magnitude higher than the 0.200 μg/m^3^ minimum risk level (MRL) for Hg^0^ as defined by the Agency for Toxic Substances and Disease Registry (ATSDR) [65]. In terms of potential for chronic toxicity, Risher notes that occupational exposure to Hg^0^ concentrations of 20 μg/m^3^ or above for several years has been linked to mild sub-clinical signs of damage to the central nervous system [66]. The United States Environmental Protection Agency’s interim Acute Exposure Guidance Limit (AEGL) does not identify an AEGL-1 for Hg^0^ as the vapor is odorless, colorless, tasteless, and at lower concentrations causes no discomfort or irritation. The 8-hour AEGL-2 is 330,000 μg/m^3^ [67]. While concentrations measured surrounding every item in the present study often exceed this concentration, it is important to note that no time-weighted averages were recorded. As demonstrated, physical contact with the surface of the amalgam releases more Hg^0^ vapor; the wearing of beaded necklaces and the action of rubbing the necklace on cloth are enough to exceed these concentrations. Similarly, the act of contacting the amalgam with a gloved hand transferred part of the amalgam to the glove. The smearing of the amalgam led to concentrations exceeding 1,000 μg/m^3^ surrounding the glove that were often higher than the amalgam itself due to increased surface area and possibly due to the disruption of the oxide layer on the surface.

In addition to the threat of Hg^0^ evolved from each item, high levels of Pb and As in each item analyzed present a potential health threat to people using or handling these objects. Again, the transfer of metals and metalloids from the amalgam to contacted surfaces can spread contamination. The offerings of milk poured onto the *shivlings* may additionally spread contamination. Apparently, some practitioners of Ayurveda will either submerge a standard *parad* object in a glass of milk or drink unboiled cow’s milk from a *parad* amrit cup in the belief that certain ailments may be cured or luck will be granted [46]. We were unable to purchase these *parad* cups for testing and were surprised that one company had stopped selling them, claiming that the quality of mercury available today is lower than that of the past. In addition, it was explained that the herbs used for the purification of Hg in the cups had gone extinct. Future studies will examine the leaching of metals from *parad* into milk.

## Conclusions

In this study, 14 *parad* items related to Hinduism and Ayurvedic medicine were demonstrated to readily evolve Hg^0^ vapor in concentrations that were orders of magnitude higher than the minimum risk level established by the ATSDR. Additionally, a simple system was used to indirectly visualize Hg^0^ vapor emitted from each object. All 14 objects were screened using a hand-held X-Ray fluorescence analyzer and determined to contain tin, arsenic and lead. Digestion and analysis of selected samples via Inductively Coupled Plasma–Optical Emission spectroscopy confirmed that each object contained high concentrations of lead and arsenic. These samples were also analyzed via direct mercury analysis and determined to exceed 20% mercury by mass. The ICP-OES and direct mercury analysis confirm that a handheld XRF can be used to screen parad items to confirm the presence of these metals. The results of this work clearly demonstrate that these objects both emit Hg^0^ vapor and are capable of easily transferring metal contamination via contact and, as such, should be handled with care. In spite of the potential for these items to spread mercury contamination and effect human and environmental health, they are exempted from the Minamata Convention on Mercury.

## Supporting information

S1 File Description and photographs of analyzed items. (DOCX)

S2 File Instrument operating parameters, performance checks, figures of merit, calibration curves. (DOCX)
