# Supplementary material for: Chemical analysis of Hg0-containing Hindu religious objects
Source: PLoS One. 2019 Dec 30;14(12):e0226855. doi: 10.1371/journal.pone.0226855 (PMC6936866; doi:10.1371/journal.pone.0226855)
Supplement: S1 File — (DOCX) [file pone.0226855.s001.docx]

**Description of Objects Analyzed**

|  | Object | Mass | Purchased From | Representative Photo |
| --- | --- | --- | --- | --- |
| 1 | Lingam | 105.9106g | Ebay.com | 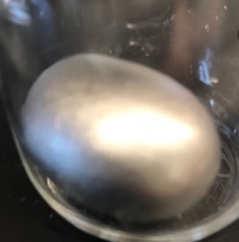 |
| 2 | Lingam | 118.6880g | Ebay.com |  |
| 3 | Lingam | 105.8539g | Ebay.com |  |
| 4 | Shivling | 9.9564g | Amazon.com | 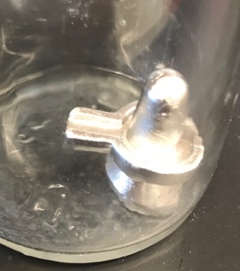 |
| 5 | Shivling | 38.8661g | Ebay.com |  |
| 6 | Shivling | 38.6787g | Ebay.com |  |
| 7 | Shivling | 21.8004g | Amazon.com |  |
| 8 | Shivling | 21.6029g | Amazon.com |  |
| 9 | Pyramid | 62.7012g | Amazon.com | 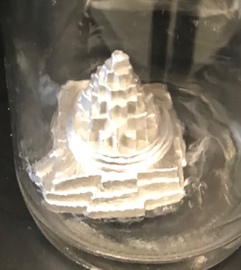 |
| 10 | Hanuman | 137.64g * | Amazon.com | 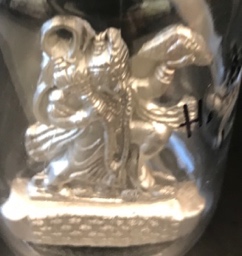 |
| 11 | Statue of God 1 | 38.0566g | Amazon.com | 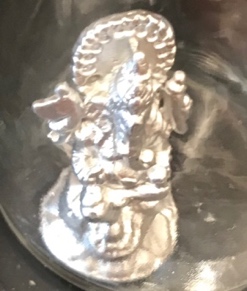 |
| 12 | Statue of God 2 | 19.5109g | Amazon.com |  |
| 13 | Statue of God 3 | 33.7205g | Amazon.com |  |
| 14 | Beads (108 beads) | ~1g/bead | Ebay.com | 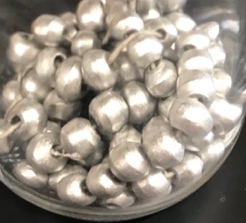 |

* Measured on a top-loading balance
