# Supplementary material for: Chemical analysis of Hg0-containing Hindu religious objects
Source: PLoS One. 2019 Dec 30;14(12):e0226855. doi: 10.1371/journal.pone.0226855 (PMC6936866; doi:10.1371/journal.pone.0226855)
Supplement: S2 File — (DOCX) [file pone.0226855.s002.docx]

**ICP-OES Operating Conditions**

A Perkin Elmer (PE) Optima 8300 Concentric ICP-OES equipped with a SC-4 DX Autosampler was used for the determination of mercury. Samples were introduced using a sea spray nebulizer with a cyclonic spray chamber. The Optima 8300, the autosampler and peristaltic pump were fully automated and controlled by the Windows ICP Syngistix Controller software. Instrument operating details for metals analysis are shown in Table 1. below.

| Parameter |  |
| --- | --- |
| RF Generator Power (W) | 1500 |
| Frequency of RF Generator (MHz) | 40 |
| Plasma Gas Flow Rate (L/min) | Ar, 10 |
| Auxiliary/Shear Gas Flow Rate (L/min) | 0.2 |
| Nebulization Gas Flow Rate (L/min) | 0.70 |
| Sample Uptake Rate (mL/min) | 1.50 |
| Type of Detector | Dual solid state |
| Injector Tube Diameter (mm) | 2.0 |
| Plasma View, distance (mm) | Axial, 15.0 |
| Read | Peak area |
| Measurement Replicates | 3 |
| Read Delay (s) | 120 |
| Rinse Time (s) | 150 (no delay) |
| Internal Standard | 10 ppm Yttrium in 2% HNO_3_ |

**Table 1. ICP-OES Instrument operating details/conditions**

A calibration curve was generated from standards ranging from 0.100-15.00 mg/L. 5.0 mL samples were analyzed via peak area using three points per peak. Yttrium was the internal standard, and samples were analyzed for Lead (Pb) at 220.353 nm, arsenic (As) at 188.979 nm, chromium (Cr) at 267.716 nm and manganese (Mn) at 257.610 nm. Instrument performance checks are shown in Table 2.

**Instrument Performance Checks: ICP – OES**

| QA/QC | Makeup | Use |
| --- | --- | --- |
| Instrument Performance Check (IPC) | 1.0 mg/L calibration solution of Pb, As, Cr, Mn | Analyzed at the beginning, after every 10^th^ sample and at the end of analysis |

**Table 2. ICP-OES Instrument Performance Checks**

**Direct Mercury Analyzer-80 Analyses:**

A DMA-80 single beam spectrophotometer with sequential flow and two measurement cells was used for the determination of Hg. The instrument used thermal decomposition of the sample followed by catalytic reduction of all mercury to Hg°, amalgamation, and atomic absorption spectrometry. The DMA-80 was automated and controlled by the Windows Easydoc 3.3 software. Instrument operating details for Hg analysis are shown in Table 3. below.

| Parameter |  |
| --- | --- |
| Typical Working Range (ng) | 1^st^ cell (low: 0-20); 2^nd^ cell: (high 20-1000) |
| Limit of Detection (ng) | 0.005 |
| Wavelength: Hg (λ, nm) | 253.65 |
| Detector | n.1 Silicon UV photodetector |
| Drying Time (s) | 30 |
| Drying Temperature (^o^C) | 200 |
| Decomposition Time (s) | 90 |
| Waiting Time (s) | 90 |
| Decomposition Temperature (^o^C) | 650 |
| Amalgamation Temperature (^o^C) | 850 |
| Amalgamation Time (s) | 12 |

**Table 3. DMA-80 Instrument operating parameters**

Because sequential cuvettes are used in DMA-80 analyses, the range of mercury calibration standards were 0.0 ng – 15 ng for the first, cell (Cell 1) used for measuring low Hg concentrations, and 30 ng – 300 ng for the second, cell (Cell 2) used for measuring high Hg concentrations. 100 $\mu$L samples were placed in quartz boats and analyzed. Since liquid-based samples were being analyzed, the sample boats were spaced two sample cells apart from one another to minimize evaporation during thermal decomposition of the previous sample. Instrument performance checks are shown in Table 4.

| QA/QC | Makeup | Use |
| --- | --- | --- |
| Instrument Performance Check (IPC): Hg calibration standard | 1.0 mg/L Hg solution diluted from a 1000 ppm Hg standard in 5% HNO_3_ | 100 $\mu$L injected directly into quartz boats and analyzed at beginning, after every 10^th^ sample and at the end of each analysis |
| Standard Reference Material (SRM) | Has a verified concentration of 1.557 mg/kg $\pm$ 0.020 | 100 $\mu$L injected directly into quartz boats and analyzed at end of sample run |

**Table 4. DMA-80 Instrument Performance Checks**

**QA/QC: Instrument Performance Check and Certified Reference Material**

Due to the variability in the samples studied, there was not a suitable standard reference material (SRM) to use for ICP-OES. However, instrument performance checks (IPC) were used throughout the analysis. An IPC was prepared similarly to the standard calibration solutions. 1.0 mg/L solutions for each metal were used. Yttrium was added as an internal standard via split-injection.

A 1.0 mg/L Hg solution was used as an IPC for the DMA-80. For the Hg analysis, which was measured using a Milestone direct mercury analyzer, a NIST mercury in water standard reference material (NIST SRM 1641d) was used. The verified concentration of the SRM was 1.557 mg/kg $\pm$ 0.020 mg/kg. In addition, an IPC was used throughout the analysis.

For both the ICP-OES and DMA analysis the IPC was measured before analysis, after every 10^th^ sample and at the end of analysis. For the DMA analysis only the SRM was measured before analysis, after every 10^th^ sample and at the end of analysis. The results from the IPC and SRM measurements are shown in the table below.

|  | **Average ICP-OES** | **Average DMA** | **% Error** |
| --- | --- | --- | --- |
|  | **IPC (1 ppm)** | **[Hg] (ppm)** |  |
| **Cd** | 1.01 ± 0.10 | ---------------------- | 0.70 |
| **Cr** | 1.06 ± 0.12 | ---------------------- | 5.90 |
| **Pb** | 1. 18 ± 0.07 | ---------------------- | 18.6 |
| **As** | 1.10 ± 0.06 | ---------------------- | 10.9 |
| **SRM (Hg in water)** | ---------------------- | 1.670 ± 0.032 | 4.4 |
| **IPC/Cal Std (1 ppm)** | ---------------------- | 1.056 ± 0.011 | 5.6 |

**Table 6. QA/QC Performance Check Data**

Since the XRF and ICP-OES data point to comparable concentrations of metals determined in the samples, these methods validate one another through direct comparison of those results determined.

**Analytical Figures of Merit.**

Figures 1-4 below are calibration curves obtained using a Perkin Elmer (PE) Optima 8300 Concentric ICP-OES equipped with a SC-4 DX Autosampler. Samples were introduced using a sea spray nebulizer with a cyclonic spray chamber. The detection limit (3σ) observed for each element is noted above each figure. The precision observed for these calibration standards is below 3% RSD.

**Cd**

LOD: 0.01 ppb

Calibration Sensitivity: 46681

Precision for Cd calibration standards: <3%

**Cr**

LOD: 0.01 ppb

Calibration Sensitivity: 46681

Precision for Cd calibration standards: <3%

**Pb**

LOD: 0.001 ppb

Calibration Sensitivity: 5100

Precision for Cd calibration standards: <3%

**As**

LOD: 0.004 ppb

Calibration Sensitivity: 1652

Precision for Cd calibration standards: <3%
